# Subcellular distribution of the rAAV genome depends on genome structure

**DOI:** 10.1038/s41598-023-44074-x

**Published:** 2023-10-13

**Authors:** Nuri Oh, Naresh H. Tarte

**Affiliations:** Department of Chemistry and Biology, Korea Science Academy of KAIST, Busan, 47162 Republic of Korea

**Keywords:** Imaging, Genetic transduction, Transfection, Transmission electron microscopy

## Abstract

Many studies have been conducted on the transduction efficiency of recombinant adeno-associated virus (rAAV) depending on the serotype and genome structure, such as single-stranded (ss) and self-complementary (sc). To understand the variation in therapeutic efficacy, we focused on investigating subcellular distribution of viral genome depending on rAAV genome structure. It is critical to ascertain the location of the virus within the host cell after the entry because a larger amount of the viral genome placed in the nucleus facilitates viral genome replication by utilizing the host cell's system, thereby enhancing the therapeutic outcome. In this sense, tracking the location of the virus within the host cell's organelles can inform a new strategy to improve therapeutic efficacy. Therefore, we attempted to stain only the viral genome with APEX2 and DAB chemicals specifically, and the distribution of the viral genome was examined by transmission electron microscopy (TEM). Consequently, when the two types of rAAV were transduced for 6 h, scAAV2 tended to be more located in the lysosome and nucleus compared to ssAAV2.

## Introduction

In recent decades, many efforts have been made to deliver nucleic acids for gene therapy using recombinant adeno-associated virus (rAAV)^1–4^. As a result of these efforts, gene therapy drugs, such as zolgensma, are commercially available to treat patients with spinal muscular atrophy. Despite these efforts, a deeper understanding of the mechanisms underlying therapeutic effects depending on rAAV genome structure is required. Support for research on this mechanism will improve the biosafety of gene therapy using rAAV. Here, ssAAV2 and scAAV2 show similar cell uptake rates, but the therapeutic effects in the central nervous system or liver tissue differ^5–8^. We believe that the frequency of viral genome distribution in subcellular organelles such as lysosomes, Golgi bodies, ER, and nucleus varies depending on the genome structure, resulting in differences in therapeutic effects^3,5,8^.

In order to support the correlation between the subcellular location of the viral genome and the therapeutic effect, we used representative methods to observe the viral genome using fluorescence microscopy and TEM. Overall, TEM has a higher resolution (~ 0.2 nm) than SEM and confocal microscopy^9^. Thus, it is a microscopy optimized for observing organisms inside cells. Furthermore, the TEM staining technique must be applied to the biological samples to examine the target viral genome. Negative and positive staining are the two most common methods for enhancing the contrast of biological samples for TEM. Both techniques focused on enhancing the contrast of the organelle’s surrounding environment using phosphotungstic acid, a heavy metal salt, and uranyl acetate, respectively^10,11^.

Although these methods have been advanced, many difficulties remain in staining only specific parts of interest. In order to overcome this limitation, many researchers developed proximity labeling with APEX2^12,13^.

In particular, the technology for specifically staining the intracellular viral genome was inadequate compared with those of viral particles. Therefore, in this paper, we first attempted to stain the viral genome using two plasmids, DD-dSpyCas9-mCherry-APEX2 (pAPEX2) and psgITR, capable of expressing dCas9-APEX2 and sgITR, respectively. Here, APEX2 has peroxidase activity which helps DAB polymerization identify the location at the subcellular level of the viral genome.

Using this technique, it was confirmed that scAAV2 was frequently located in the nucleus compared to ssAAV2 and is more likely to have its viral genome placed in the lysosome.

## Results

### Optimization of transfection condition for viral genome staining

**Figure 1:**
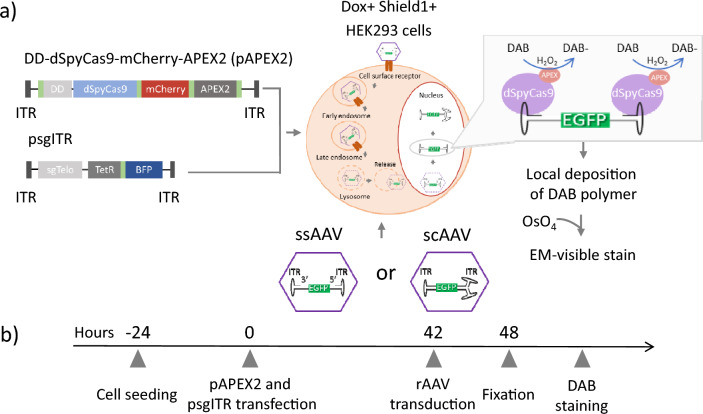
The experimental procedure to stain viral genome. (**a**) The DD-dSpyCas9-mCherry-APEX2 (pAPEX2) expressed and single guide RNA for ITR (psgITR) expressed plasmids constructions were transfected to HEK293 cells and showing how DAB polymerization by APEX2 near viral genome was used as reporters to make a contrast for TEM. DAB, diaminobenzidine. (**b**) Schematic of the experimental procedure for proximity labeling with APEX2 and DAB. “p” indicates plasmid. “ss” before AAV represents single-stranded, “sc” before AAV designates self-complementary.

The transfection was prepared by various concentrations of plasmids expressing DD-dSpyCas9-mCherry-APEX2 (pAPEX2) and sgITR (psgITR) with the transfection agent to stain the rAAV genome inside the cells for TEM. To investigate the target viral genome, a specific staining technique is necessary for the sample materials. The enhanced ascorbate peroxidase 2 (APEX2) can be used to label intracellular proteins for imaging by electron microscopy^13^. In order to stain only the viral genome, we used a plasmid encoding dCas9-mCherry-APEX2 and designed a BFP-sgITR to assist the dCas9-APEX2 protein complex in locating the ITR region of the viral genome. Furthermore, by adding DAB chemicals, APEX2 made polymerized DAB for local deposition near the ITR region so that the viral genome could be observed by TEM (Fig. 1a).

In order to deliver pAPEX2 and psgITR, we used Mirus (Mirus Bio LLC, USA), which is known to carry plasmid DNA and siRNA to the cell via the endocytosis pathway and escape from endosomes for cytosolic delivery of both plasmid DNA and siRNA^14,15^. HEK293 cells are widely used to produce rAAV^15,16^. Transfection was carried out using the following procedures: incubation of the complexes consisting of pAPEX2, psgITR expressing mCherry and blue fluorescence protein respectively, and Mirus for 42 h and subsequent incubation of the 4000 MOI rAAV for 6 h (Fig. 1b).

**Figure 2:**
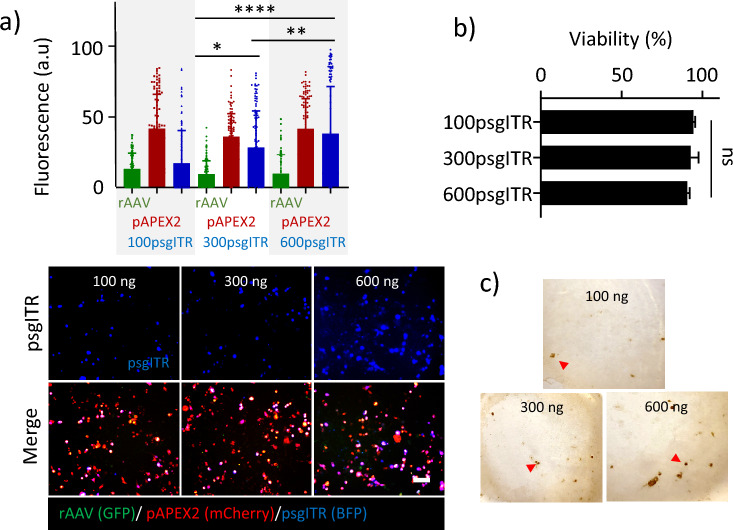
The optimal condition of transfection for proximity labeling with pAPEX2 and psgITR. (**a**) Quantification of fluorescence after transfection of pAPEX2 and psgITR, transduction of rAAV (top), the corresponding images of fluorescence microscopy of the cells (bottom), (**b**) cytotoxicity of transfection agents prepared at two plasmids and rAAV. (**c**) proximity labeling using APEX2 and DAB with different amounts of psgITR. Dark reaction products indicate DAB polymerization by APEX2 (arrow). Data represent means ± SD. Statistical significance was analyzed by one-way analysis of variance, followed by Turkey’s test [n = 3, * *p* < 0.05, ** *p* < 0.01, and *** *p* < 0.001], the scale bar represents 100 µm.

In order to optimize the amount of psgITR for efficient DAB staining, we treated various amounts of psgITR, 100, 300, and 600 ng with 100 ng of pAPEX2 and 4000 MOI rAAV per 500,000 cells. The transfection efficiency (TFE) of the plasmids and the transduction efficiency (TDE) of each rAAV were examined by fluorescence microscopy and quantified by Image J open source software on a single-cell basis (Fig. 2a), and DAB polymerization by APEX2 was investigated with bright microscopy (Fig. 2c). Based on this analysis, 600 ng of psgITR significantly showed the highest transfection efficiency (BFP) with low cytotoxicity (Fig. 2a and b). Additionally, it was found that the fluorescence signal of pAPEX2 (mCherry) and rAAV (GFP) showed constant expression levels of each protein regardless of the increase in the amount of psgITR. As shown in Fig. 2c, cells were fixed with 2% (vol/vol) glutaraldehyde; after DAB staining with APEX2, a DAB polymerization product was visible by bright microscopy. A combination of 100 ng of pAPEX2, 600 psgITR, and 4000 MOI rAAV showed more dark reaction products compared to other experimental groups corresponding with a TFE of psgITR. Based on this result, we kept the above transfection condition for DAB staining with APEX2 to investigate the viral genome by TEM.

### Investigation of the viral genome using DAB staining by APEX2

**Figure 3:**
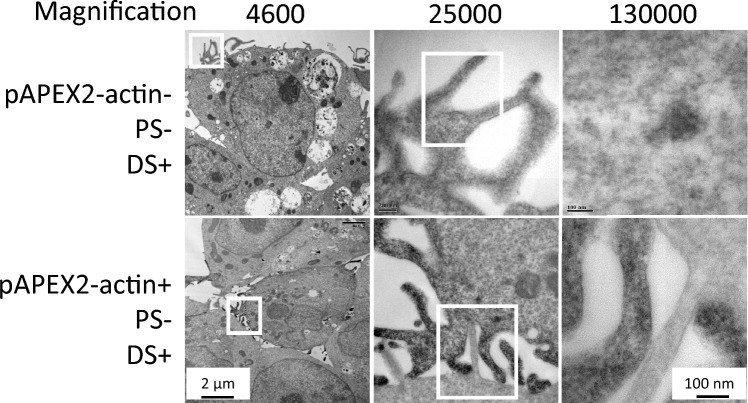
Observation of actin filament with APEX2 and DAB staining in HEK293 cells. Transmission electron microscopic (TEM) images DAB stained actin filament in HEK293 cells. The cells were treated with plasmids capable of expressing APEX2 around actin for 48 h. The magnified images on the right side come from the squares in the corresponding images on the left side.

Before comparing the differences in subcellular distribution depending on the genome construction of rAAV, we carried out the experiment to verify TFE and TDE with psgNS and psgITR. ssAAV2 and scAAV2 were incubated with pAPEX2 and psgITR for viral genome targeting, and both rAAVs were incubated with pAPEX2 and psgNS to prepare nonspecific targeting as the negative control. When we compared TFE between the groups of pAEX2 and psgITR or pAPEX2 and psgNS, there were no significant differences in TFE regardless of the type of psgRNA and genome construction of rAAV (Supplementary Fig. 1). Based on the result of TFE and TDE, we proceeded only with DAB staining. It indicated that we did not carry out negative and positive staining, which is, in general, staining methods for TEM in addition to DAB staining to observe the viral particles or genome with DAB polymer more clearly. Both methods are non-targeting staining, making it difficult to distinguish between the organelle structure and DAB polymerization near the viral genome. Furthermore, to more accurately confirm whether DAB staining was successful or not in terms of precise staining of targeted area, 100 ng of APEX2-actin plasmids capable of expressing APEX2 near actin filaments was transfected as a technical control, and DAB staining was performed. As shown in Fig. 3, it was confirmed by high magnification observation that actin performed well in DAB staining compared to the control in which the APEX2-actin plasmid was not transfected. High-magnification observation is required to observe DAB staining because they appear to be similar to ribosomes or condensed chromatin when observed under low magnification with TEM.

### Distribution of viral genome depending on genome structure

**Figure 4:**
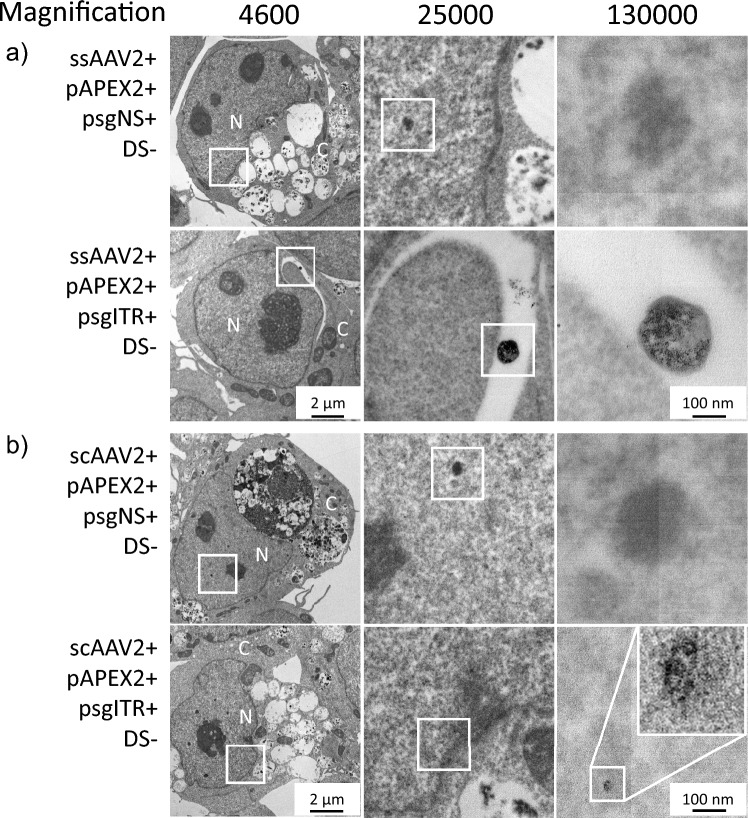
Investigation of ssAAV2 and scAAV2 genome distribution in HEK293 cells. (**a**) and (**b**) Transmission electron microscopic (TEM) images of DAB stained ssAAV2 (**a**) and scAAV2 (**b**) genome in HEK293 cells. HEK293 cells were pre-treated with the complex consisting of pAPEX2, psgITR, and Mirus for 42 h and then further incubated in virus-containing media for 6 h. After post-incubation, the cells were fixed, stained, and examined by TEM to visualize DAB stained viral genome. The magnified images on the right side come from the squares in the corresponding images on the left side. The letters PS and DS indicate positive staining and DAB staining respectively. N and C designate the nucleus and cytoplasm, respectively.

Based on the TEM images, we investigated DAB polymerization at the highest magnification of TEM (Fig. 4a and b).

**Figure 5:**
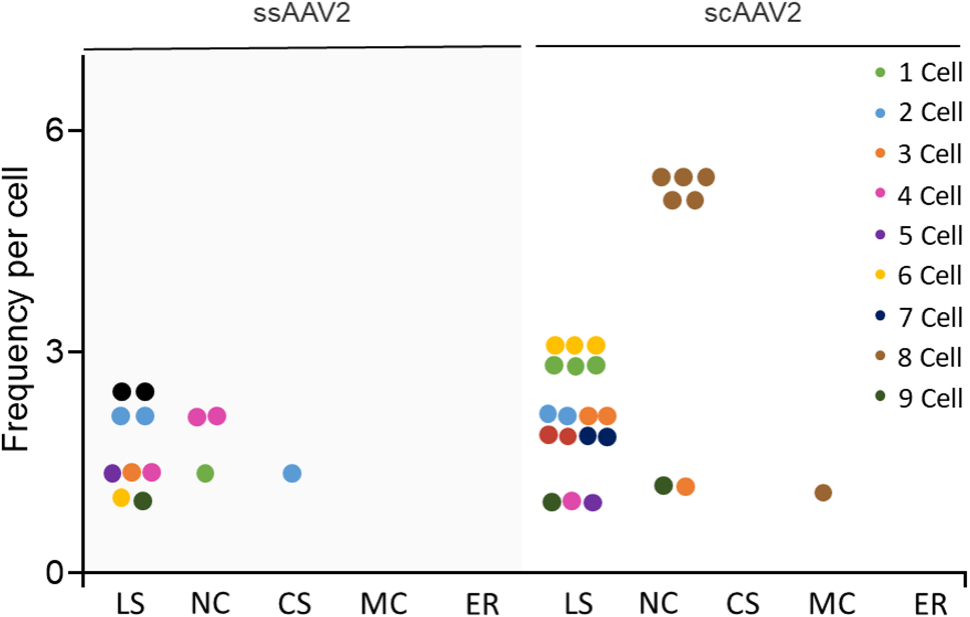
Frequency comparison of discovery of viral genomes between ssAAV2 and scAAV2 interrogated by DAB polymers. LS: lysosome, NC: Nucleus, CS: Cytoplasm, MC: Mitochondria, ER: Endoplasmic reticulum.

In general, rAAV is known to enter cells through the endocytosis pathway, and only the virus genome enters the nucleus after endosome escape^17,18^. To observe the distribution of viral genome at subcellular level, we identified the nucleus, lysosome, mitochondria, and endoplasmic reticulum based on their size and shape^12,19,20^ and DAB polymerizations representing the viral genome were counted from nine cell images obtained from ssAAV2 and scAAV2, respectively. As a result, the viral genome was found fundamentally in the nucleus and lysosome. It was more located in the lysosome than in the nucleus, regardless of rAAV genome structure (Fig. 5). However, in the case of scAAV2, the frequency found in the lysosome and nucleus was approximately twice as high as that of ssAAV2. Representative TEM images show how DAB polymers were distributed near the ITR region of the virus in the nucleus and lysosome, respectively (Fig. 4). This result is considered to be related to therapeutic efficacy.

## Discussion

Since the rAAV genome size is known to be ~ 4.7 kilobases (kb) long^21^, special equipment with very high resolution is needed to investigate the individual location of viral genomes in host cells due to their size. Electron microscopy (EM) has a superior spatial resolution (~ 1 nm) compared to those of fluorescence microscopy (200–300 nm)^22^. In addition to this resolution issue, the staining method to target viral genome of interest is required to determine their location within the cell. Therefore, we used the APEX2 gene tag, which can efficiently cause DAB polymerization by APEX2 at 4 °C^12^. By adding osmium tetroxide (OsO4), this polymer becomes EM-visible near target materials. In addition to the function of DAB, the DAB polymer is well-preserved and shows local deposition in the specific region of interest in immunoperoxidase labeling^23^. Although there are several advantages to EM, a staining method using genetic tags has an issue regarding off-targeting. To compensate for this disadvantage, we introduced the dCas9-APEX2 construct and sgITR respectively which was able to confirm that dCas-APEX2 can be directed to nearly any genomic region of interest by sgITR, and then the enzymatic activity of APEX2 was verified with DAB polymerization that appears as a dark particle.

In addition to this issue, cells contain numerous structures that resemble DAB polymers. At low magnification, ribosomes, condensed chromatin, and microfilaments with cross-sections are particularly difficult to distinguish from DAB polymers. In addition, the nucleus and lysosomes are more likely to contain viral genomes than other subcellular organelles, but DAB polymers are difficult to observe in the nucleus compared to the lysosome due to differences in the area and composition of these organelles.

In other words, even if the same amount of viral genome is in the nucleus and lysosome, the area of the nucleus is relatively larger than the lysosome and is densely packed with condensed chromatin. Thus, the viral genome appeared to be scattered across the nucleus, and the cross-sectioned condensed chromatin appears to be similar to the DAB polymer that stained the viral genome when investigated at low magnification because the electron density of both constructions is similar.

In order to distinguish it from other subcellular organelles, the target viral genome can be visualized using DAB polymer. The ultra-fine structure of the DAB polymer must be examined under high magnification. Lastly, such work should continue to ensure the safety of the virus as a drug, and this research paves the way for rAAVs’ increased clinical translation.

## Methods

### Cell culture

Human embryonic kidney 293 (HEK293) provided by Dr. Park. was maintained in Dulbecco’s modified Eagle medium (DMEM, Life Technologies) supplemented with 10% FBS (Sigma), 100 μg/mL penicillin, and streptomycin in a 37 °C humidified incubator (5% CO_2_, 95% air).

### Vector construction

Green fluorescent protein (GFP) expressing self-complementary recombinant adeno-associated virus type 2 (scAAV2) and single-stranded recombinant adeno-associated virus type 2 (ssAAV2) rAAV plasmids expressing were donated from Vector Core (Worcester, MA, USA). We use the Shield1- and doxycycline-inducible dSpyCas9–mCherry–APEX2 construct into DD–dSpyCas9–mCherry^24^ using the pHAGE backbone, and the sgITR-encoding construct by replacing the C3guide RNA sequence (pCMV_C3_sgRNA_2XBroccoli/pPGK_TetR_P2A_BFP) with sgITR sequences. sgNS for nonspecific targeting as control was constructed similarly. We used APEX2-actin (Addgene # 66172) as technical control.

### Plasmid transfection and rAAV infection

HEK293 cells were seeded at a density of 5.0 × 10^5^ cells/well in a 6-well plate and cultured in a medium for 24 h. The APEX2 construct and the plasmids expressing sgRNA were mixed with Mirus (Mirus Bio LLC, USA) according to the manufacturer’s instructions and introduced into cells for 42 h when they had grown to a confluence of 60 to 90%. Approximately 42 h after transfection of two plasmids, ssAAV2 and scAAV2 were infected for 6 h in HEK293 cells. A well was prepared to transfect with a negative-control construct (lacking APEX2) using the same transfection procedure. Another well was prepared to transfect with the APEX2 construct known to produce strong DAB staining, such as APEX2-NES (Addgene, cat. no. 49386) or APEX2-actin (Addgene, cat. # 66172) as a positive control.

### Sample preparation for TEM

After rAAV infection, all cell media were removed by aspiration, and immediately add 250 μL of a warm (30–37 °C) 2% (vol/vol) glutaraldehyde solution by gentle pipetting. The cells were incubated at room temperature for 5 min and then placed on ice for 60 min. The cells were washed three times for ~ 1 min each time in 250 μL of cold (0–4 °C) 1 × sodium cacodylate, and then washes were performed gently, removing the liquid from the cells by aspiration. 250 μL of cold (0–4 °C) 20 mM glycine solution was added to cells for 5 min on ice and then removed by gentle aspiration and washed three times for 1 min each time in a cold buffer. After removing the buffer, 250 μL of 1 × DAB solution with 10 mM H_2_O_2_ was added for 5–45 min until a light brown stain was visible under a stereo light microscope, and then the DAB solution was removed and washed three times for 1 min each time in 1 × sodium cacodylate. Cells were pelleted via centrifugation at 1,000 rpm for 5 min and fixed with 2.5% glutaraldehyde (Sigma) for 1 day at 4 °C. The pellet was then incubated in 1% osmium tetroxide (OsO4, Sigma) for 1 h, dehydrated in a series of alcohol, and substituted with 100% propylene oxide. The cells were then embedded in the solution comprising propylene oxide and Epon812 for 24 h and solidified at 70 °C. Finally, the cells are sliced to a thickness of 70 nm and imaged by TEM (FE-TEM, JEOL).

### Cytotoxicity assay

A total of 5.0 × 10^5^ cells of HEK293 cells were seeded in 6-well plates with ∼90% confluence. 100 ng, 300 ng, and 600 ng psgITR with 100 ng pAPEX2 were treated in HEK293 cells for 42 h in serum-supplemented media at 37 °C incubator (5% CO_2_, 95% air) and then 4000 MOI rAAV was further incubated in the cells for 6 h. The cells were gently washed and examined by the colorimetric thiazolyl blue tetrazolium bromide assay (Sigma).

### Statistical analyses

Experimental and control data represent the mean ± standard deviation (SD). Statistical differences were analyzed by one-way analysis of variance, followed by Tukey’s test using Prism v8.0 software (GraphPad, La Jolla, CA, USA). *p* values < 0.05 were considered significant (*p < 0.05, **p < 0.01, and ***p < 0.001).

### Supplementary Information

Supplementary Figure 1.

## Data Availability

The datasets used and/or analyzed during the current study are available from the corresponding author on reasonable request.
